# Mutation of CD2AP and SH3KBP1 Binding Motif in Alphavirus nsP3 Hypervariable Domain Results in Attenuated Virus

**DOI:** 10.3390/v10050226

**Published:** 2018-04-27

**Authors:** Margit Mutso, Ainhoa Moliner Morro, Cecilia Smedberg, Sergo Kasvandik, Muriel Aquilimeba, Mona Teppor, Liisi Tarve, Aleksei Lulla, Valeria Lulla, Sirle Saul, Bastian Thaa, Gerald M McInerney, Andres Merits, Margus Varjak

**Affiliations:** 1 Institute of Technology, University of Tartu, 50411 Tartu, Estonia; m.mutso@griffith.edu.au (M.M.); sergo.kasvandik@ut.ee (S.K.); muriel.aquilimeba@hotmail.fr (M.A.); mona.teppor@ut.ee (M.T.); tarve.liisi@gmail.com (L.T.); aleksei.lulla@gmail.com (A.L.); leralulla@gmail.com (V.L.); sirlesaul@gmail.com (S.S.); 2 Department of Microbiology, Tumor and Cell Biology, Karolinska Institutet, SE-171 77 Stockholm, Sweden; ainhoa.moliner.morro@ki.se (A.M.M.); Cecilia.Smedberg@ki.se (C.S.); bthaa@gmx.de (B.T.); Gerald.McInerney@ki.se (G.M.M.)

**Keywords:** alphavirus, Chikungunya virus, CD2AP, SH3KBP1, SH3 ligand, proteomics

## Abstract

Infection by Chikungunya virus (CHIKV) of the Old World alphaviruses (family Togaviridae) in humans can cause arthritis and arthralgia. The virus encodes four non-structural proteins (nsP) (nsP1, nsp2, nsP3 and nsP4) that act as subunits of the virus replicase. These proteins also interact with numerous host proteins and some crucial interactions are mediated by the unstructured C-terminal hypervariable domain (HVD) of nsP3. In this study, a human cell line expressing EGFP tagged with CHIKV nsP3 HVD was established. Using quantitative proteomics, it was found that CHIKV nsP3 HVD can bind cytoskeletal proteins, including CD2AP, SH3KBP1, CAPZA1, CAPZA2 and CAPZB. The interaction with CD2AP was found to be most evident; its binding site was mapped to the second SH3 ligand-like element in nsP3 HVD. Further assessment indicated that CD2AP can bind to nsP3 HVDs of many different New and Old World alphaviruses. Mutation of the short binding element hampered the ability of the virus to establish infection. The mutation also abolished ability of CD2AP to co-localise with nsP3 and replication complexes of CHIKV; the same was observed for Semliki Forest virus (SFV) harbouring a similar mutation. Similar to CD2AP, its homolog SH3KBP1 also bound the identified motif in CHIKV and SFV nsP3.

## 1. Introduction

The alphaviruses (family *Togaviridae*) are widely spread all over the globe, found on every continent, and have been identified not only in different vertebrate hosts such as mammals, birds and fish, but also in insects. There are several human and animal pathogens in the alphavirus genus [1,2]. The majority of alphaviruses are transmitted between different vertebrate species by various mosquitoes [1,3,4]. Mosquito-transmitted alphaviruses can be divided into Old and New World alphaviruses. Chikungunya virus (CHIKV), belonging to the Old World alphaviruses, has relatively recently emerged in Asia, Africa and America and its infection results in severe illness in humans [5]. Semliki Forest virus (SFV) and Sindbis virus (SINV) also belong to Old World alphaviruses and have been extensively used as models for studies of alphaviruses. Most studied of the New World alphaviruses are Venezuelan equine encephalitis virus (VEEV), Eastern equine encephalitis virus (EEEV) and Western equine encephalitis virus (WEEV) whose infections have economic and medical consequences in South and Central America. Despite the similarity of genome organization and basic replication strategy of Old and New World alphaviruses, these groups greatly differ in their host interactions, e.g., use different strategies to counteract host anti-viral responses and interact with a different set of host proteins [6,7]. Significant differences in virus-host interactions have also been observed for alphaviruses belonging to the same group, for example SFV and CHIKV [8,9] or VEEV and EEEV [7,10].

The alphavirus genome is a single-stranded RNA molecule with positive polarity, its 5′ end is capped and the 3′ end has a poly(A) tail. In infected cells, genomic RNA is used as an mRNA for the translation of the large non-structural (ns) polyprotein P1234, the precursor of four mature replicase proteins nsP1, nsP2, nsP3 and nsP4 [3,11]. Alphavirus RNA replication depends on P1234 expression and its highly-regulated processing. The early viral replication complex, which consists of the P123 processing intermediate and mature nsP4, synthesizes negative strand RNA using the genomic strand as template, resulting in the formation of double-stranded RNA (dsRNA) replication form [12,13]. This process coincides with the formation of plasma membrane invaginations termed spherules that contain viral dsRNA, nsPs and cellular components [14,15]. The processing of the P123 intermediate into mature nsP1, nsP2 and nsP3 brings an end to the synthesis of negative-strand RNA and triggers formation of late replication complexes which use viral dsRNA template to make new genomic and subgenomic RNA strands [12,16]. The latter is synthesized from an internal promoter located on the negative strand RNA and its sequence corresponds to that of the last one third of the genomic RNA. Subgenomic RNA is needed for the synthesis of alphavirus structural proteins C, E3, E2, 6K, TF and E1 [17,18,19,20,21].

In infected vertebrate cells, alphavirus spherules are first observed at the plasma membrane; later in infection they are often re-localised to internal membranes, such as endosomal or lysosomal membranes [11,14,15]. nsP1 anchors the replication complexes to the membranes [22,23], and is involved in the synthesis of cap-structure and RNA negative strands [24]. nsP2 has protease activity, which is required to process P1234 into mature non-structural proteins [25,26]. In addition, nsP2 is also an RNA triphosphatase involved in viral cap synthesis and has NTPase and RNA helicase activities [27,28,29]. nsP4 is the viral RNA-dependent RNA polymerase and terminal adenosine transferase and is directly responsible for synthesis of different viral RNAs and adding poly(A) tails to positive strand RNAs [30,31,32].

For a long time, the roles of nsP3 in virus infection were poorly understood. However, recent data have revealed nsP3 involvement in different alphavirus-host interactions. nsP3 can be divided into three domains: N-terminal macro domain, middle conserved domain termed alphavirus unique domain (AUD) or zinc-binding domain, and C-terminal hypervariable domain (HVD) [7,33,34,35]. 3D structures have been revealed for the first two of these domains while the HVD has been predicted to be intrinsically unstructured [33,34]. The macro domain can bind RNA, mono(ADP-ribose) and poly(ADP-ribose). For some alphaviruses the macro domain possesses adenosine diphosphoribose 1”-phosphate phosphatase activity, which likely gives the ability to hydrolyze ADP-ribose groups from aspartate and glutamate residues of mono(ADP-ribosyl)ated proteins [33,34,36]. Similarly, the macro domain of distantly related hepatitis E virus can remove mono(ADP-ribose) but also poly(ADP-ribose) from proteins [37]. This function is crucial for alphavirus infection both in vertebrate and invertebrate cells [38]. In addition, the macro domain also coordinates P1234 processing [33]. Many mutations in AUD are lethal for alphaviruses indicating that this region is also essential for virus infection. The functional roles of the AUD are, however, unknown.

As the name implies, the HVD is poorly conserved: with the exception of scattered short linear motifs, there is little similarity between HVDs of different alphaviruses [7,35,39]. For several alphaviruses, such as SFV and SINV, it is known that HVD is phosphorylated [40,41], the same is presumed for other alphaviruses as well. HVD is an intrinsically unstructured region, tolerates large insertions and deletions and has been shown to bind various host proteins [39,42,43,44,45]. Indeed, binding motifs for several of such host proteins have already been identified within the domain. HVD of Old World alphaviruses bind Ras GTPase-activating protein-binding protein 1 (G3BP1) and Ras GTPase-activating protein-binding protein 2 (G3BP2) using a duplicated FGDF motif located almost at the very end of the domain, just before the degradation signal [39,46,47]. Unlike many other interactions with host proteins, the nsP3:G3BP interaction is crucial: in the absence of these proteins (or if the interaction motifs are removed) the Old World alphaviruses replicate poorly or not at all [7,48,49]. In addition, the nsP3:G3BP interaction disrupts stress granule formation that may also be beneficial for virus infection [46]. nsP3 of New World alphaviruses bind different stress granule components Fragile X mental retardation 1 (FMR1), FMR1 autosomal homolog 1 (FXR1), and FMR1 autosomal homolog 2 (FXR2); this interaction is also required for efficient virus replication [7]. Curiously, EEEV occupies the “middle ground” and can utilize both G3BP and FXR proteins to support its replication [10]. Milder effects on alphavirus infection are observed if interactions with other host proteins are prohibited. Examples of such proteins include amphiphysins that bind via their SRC Homology 3 (SH3) domains to the polyproline-rich nsP3 region of the Old World alphaviruses [50]. In addition, deletion of phosphorylated regions of SFV nsP3 HVD disrupted the hyper-activation of the pro-survival PI3K-Akt-mTOR pathway. Further analysis indicated the presence of a YXXM motif that is needed for binding of PI3K regulatory subunit p85, which results in Akt activation [8,51]. The many of functions of the enigmatic alphavirus nsP3 proteins have been recently reviewed by Gotte et al., 2018 [52].

To better understand the role of HVD in binding host cell proteins and its importance for alphavirus infection, a proteomics study was performed to systematically identify binding partners of the CHIKV nsP3 HVD. Among other proteins, CD2-associated protein (CD2AP) and SH3 domain-containing kinase-binding protein (SH3KBP1) were identified as prominent interaction partners. Both proteins co-localised with CHIKV and SFV replication complexes in infected cells. The binding site was mapped to the HVD and point mutations in this site were shown to disrupt interaction with CD2AP and SH3KBP1 as well as several functionally connected cellular proteins. The motif was found to be common, yet not universal, for both Old and New World alphaviruses. The mutant viruses lacking the motif for CD2AP and SH3KBP1 binding were infectious, albeit attenuated. Our findings indicate that the nsP3 interactions with CD2AP and SH3KBP1 (and/or interaction with proteins interacting with the same binding motif) is required for efficient infectivity of alphaviruses, but has no major impact on localisation of viral replication complexes.

## 2. Materials and Methods

### 2.1. Cell Lines

BHK-21 (baby hamster kidney, ATCC CCL-10) cells were grown in *Glasgow minimum essential medium* (GMEM, Thermo Fisher Scientific, Waltham, MA, USA) containing 10% fetal bovine serum (FBS, Thermo Fisher Scientific), 2% tryptose phosphate broth (TPB), 20 mM HEPES and penicillin-streptomycin (100 U/mL and 0.1 mg, respectively). HOS (human osteosarcoma, ATCC CRL-1543) cells were cultured in Dulbecco’s modified Eagle’s *medium* (DMEM, Thermo Fisher Scientific) supplemented with 10% FBS, 2mM l-glutamine, and penicillin-streptomycin. For cultivation of Flp-In T-REx 293 cells (Thermo Fisher Scientific) DMEM also contained zeocin (at 100 μg/mL) and blasticidin (at 15 μg/mL). For propagation of transgenic cell lines based on Flp-In T-REx 293 zeocin was omitted and hygromycin B (at 100 μg/mL) was used instead. All the cells were grown in a humidified incubator at 37 °C in a 5% CO_2_ atmosphere.

For proteomics study cell lines were labelled with stable isotopes as follows: cells were grown in SILAC DMEM (Thermo Fisher Scientific) supplemented with 10% dialyzed FBS (Thermo Fisher Scientific), heavy arginine (0.133 mM, CNLM-539; Cambridge Isotope Laboratories Inc., Tewskbury, MA, USA) and heavy lysine (0.266 mM, CNLM-291; Cambridge Isotope Laboratories Inc.), hygromycin B (at 100 μg/mL), blasticidin (at 15 μg/mL) and penicillin-streptomycin for at least five generations.

### 2.2. Construction of Expression Constructs for HVD Domains of Different Alphaviruses and Making of the Stable Transgenic Cell Lines

Sequences encoding Flag-tagged EGFP and Flag-tagged EGFP fused to nsP3 HVD from CHIKV of East/Central/South African genotype isolate LR2006-OPY1 (amino acid residues 327–530 of nsP3) or VEEV (residues 327–557 of nsP3) were cloned into modified pEF5/FRT/V5-DEST vector (Thermo Fisher Scientific) under the human EF-1α promoter. Sequences encoding for HVD regions of SINV (Toto1101, residues 327–556 of nsP3), O’nyong’nyong virus (ONNV) (residues 327–549 of nsP3), Barmah Forest virus (BFV) (residues 326–470 of nsP3) and Ross River virus (RRV) (T48 strain, residues 328–535 of nsP3) were cloned using the same approach.

Point mutations preventing interaction with CD2AP (P423A, R428A for CHIKV, and P428A, R433A, P435A, P439A, R440A, R444A for SFV) as well as various deletions were introduced into HVD region of nsP3 using PCR-based site-directed mutagenesis and subcloning procedures. The obtained fragments harbouring desired mutations were transferred to infectious cDNA (icDNA) clones of CHIKV or SFV4 as well as into expression constructs used for stable or transient expression of EGFP tagged with nsP3 HVD of CHIKV or SFV. All constructs were verified using Sanger sequencing, their sequences are available upon request.

To make stable transgenic cells lines 10^7^ Flp-In T-REx 293 cells were co-transfected with 9 µg of pOG44 plasmid (Thermo Fisher Scientific) used for expression of Flp recombinase and 1 µg of expression construct for Flag-tagged EGFP, Flag-tagged EGFP fused to nsP3 HVD of VEEV, wild-type (wt) CHIKV, mutant CHIKV (P423A and R428A substitutions) or mutant SFV (P428A, R433A, P435A, P439A, R440A and R444A substitutions) using Lipofectamine 2000 reagent (Thermo Fisher Scientific). At 48 h post-transfection (p.t.) the medium was replaced with a selection medium containing hygromycin B (at 100 μg/mL); subsequently medium was changed every 3–4 days to remove dead cells. Formed hygromycin B resistant colonies were picked up and verified for the expression of intended proteins using fluorescent microscopy (for EGFP auto-fluorescence) and western blotting.

### 2.3. Virological Methods

Plasmids containing wt or mutant icDNA of CHIKV were linearized using *Not*I restriction enzyme. In vitro transcription was carried out using linearized plasmids as templates and the mMESSAGE mMACHINE SP6 transcription kit (Thermo Fisher Scientific); obtained RNA was controlled for its quality and quantified using agarose gel electrophoresis and staining with ethidium bromide.

Virus recovery was performed as follows, 8 × 10^6^ BHK-21 cells resuspended in ice-cold PBS were transfected with 10 μg of in vitro transcribed RNAs by electroporation using a Gene Pulser II unit (two pulses at 850 V and 25 μF, Bio-Rad, Hercules, CA, USA), 48 h later virus stock was harvested to be used in further experiments. Infectious centre assay (ICA) was conducted to measure RNA infectivity, 1 μg of in vitro transcribed RNA was used and ten-fold dilutions of electroporated cells were seeded on top of 1.5 × 10^6^ BHK-21 cells grown in the wells of a 6-well tissue culture plate. After 2 h of incubation at 37 °C, the cell culture medium was removed and the cells were overlaid with 2 mL of GMEM containing 0.8% carboxymethyl cellulose (Sigma-Aldrich, St. Louis, MO, USA) supplemented with 2% FBS. Cells were stained with crystal violet after 3 days of incubation at 37 °C. To recover SFV4 and its mutants pCMV-SFV4 plasmid was used as previously described [53]. Electroporated cells were plated on 6 cm diameter plates and covered by growth medium. P_0_ virus stocks we collected upon development of cytopathic effects.

The titres of obtained virus stocks and growth curve samples were determined using standard plaque assays, titre given in plaque forming units per mL (PFU/mL). For this ten-fold serial dilutions of collected stocks were prepared and used to infect BHK-21 growing in wells of 6-well plates at 90–100% confluency. 1 h post-infection (p.i.) infectious media were aspirated and cells were overlaid with 2 mL of GMEM containing 2% FBS and 0.8% carboxymethyl cellulose. Cells were fixed 3 days p.i., stained with crystal violet and plaques were counted.

### 2.4. Immunoprecipitation and Proteomics Analysis

3 × 10^7^ cells were harvested for proteomics studies; 10^6^ cells were used for co-immunoprecipitation experiments with alphavirus HVD-tagged EGFP. Cells were centrifuged and washed once with cold PBS. Harvested cells were lysed with ice-cold lysis buffer (150 mM NaCl, 20 mM Hepes (at pH 7.2), 100 mM K-acetate, 2 mM MgCl_2_, 0.1% Tween-20, 1% Triton X-100, 1 Protease Inhibitor Cocktail Tablet (Roche, Basel, Switzerland) per 50 mL) on ice for 30 min, non-lysed material was removed by centrifugation at 15,000× *g* for 20 min at 4 °C; the obtained supernatant was transferred into a new tube. Magnetic beads for binding EGFP (GFP-trap M, Chromotek, Planegg, Germany) were equilibrated with lysis buffer and added to the supernatant and incubated at 4 °C for 1 h keeping the tubes constantly rotating. The beads were then washed four times with the lysis buffer and material bound to beads was eluted by boiling in 1% SDS solution (for proteomics samples) or in 1× Laemmli buffer (for immunoblot analysis). For proteomics samples, proteins were precipitated with 10% TCA and resolubilized in 7 M urea/2 M thiourea, 100 mM ammonium bicarbonate (ABC) buffer. After disulphide reduction with 5 mM dithiothreitol and cysteine alkylation with 10 mM iodoacetamide, proteins were in-solution digested for 4 h at room temperature with 1:50 (enzyme to protein) Lys-C (Wako, Richmond, VA, USA, diluted 5-times with 100 mM ABC, and further digested overnight with 1:50 dimethylated trypsin (Sigma-Aldrich). Samples were desalted on an in-house made C-18 tips. Peptides were separated on an Ultimate 3000 RSLCnano system (Dionex, Sunnyvale, CA, USA) using a C18 cartridge trap-column in a backflush configuration and an in-house packed (3 µm C18 particles, Dr Maisch, Ammerbuch, Germany) analytical 50 cm × 75 µm emitter-column (New Objective, Woburn, MA, USA). Peptides were eluted at 200 nL/min with an 8–40% of B 90 min gradient (buffer B: 80% acetonitrile + 0.1% formic acid, buffer A: 0.1% formic acid) to a Q Exactive Plus (Thermo Fisher Scientific) tandem mass spectrometer operating with a top-5 strategy and with a maximum cycle time of 0.9 s. Briefly, one 350–1400 m/z MS scan at a resolution of R = 70,000 was followed by higher-energy collisional dissociation fragmentation (normalized collision energy of 26) of the 5 most-intense ions (z: +2 to +6) at R = 17,500. The MS and MS/MS ion target values and injection times were 3 × 10^6^, 50 ms and 5 × 10^4^, 110 ms respectively. Dynamic exclusion was limited to 25 s. Obtained raw LC/MS/MS data was identified and quantified using the MaxQuant software package [54]. Arg10 and Lys8 were defined as the heavy amino acids and spectra were searched against UniProt (http://www.uniprot.org) *Homo sapiens* reference proteome supplemented with CHIKV and SFV nsP3 sequences. Minimally two peptides at least seven amino acids long were required for identification of a protein. All other parameters were default. Two-fold difference from mean was taken as a cut-off value. Functional analysis of host proteins was conducted using the STRING database (string-db.org).

Immunoprecipitation of CHIKV or SFV nsP3 protein from infected cells was performed as described before [46]. Briefly, 2.4 × 10^6^ cells were washed with ice-cold PBS and lysed with ice-cold lysis buffer (20 mM HEPES, pH 7.4, 110 mM potassium acetate, 2 mM MgCl_2_, 0.1% Tween 20, 1% Triton X-100, 0.5% sodium deoxycholate, 0.5 M NaCl) supplemented with protease inhibitor on ice for 10 min. Cell debris was removed from the lysates by centrifugation at 14,000× *g* for 10 min at 4 °C; the obtained supernatant was transferred to a new tube and divided into two fractions, 30 µL for whole-cell lysates and 550 µL for co-immunoprecipitation assays. For co-immunoprecipitation experiments, supernatant was incubated with primary antibody (rabbit anti-SFV or anti-CHIKV nsP3, both in house) for 15 min at room temperature in constant rotation. Magnetic beads (Protein G, GE Healthcare, Chicago, IL, USA) were equilibrated with lysis buffer, added to the supernatant and incubated overnight at 4 °C in constant rotation. The beads were then washed four times with lysis buffer and proteins were eluted by heating in 1× NuPAGE LDS sample buffer (Thermo Fisher Scientific) supplemented with DTT for 10 min at 80 °C.

### 2.5. Immunoblot Analysis

Protein samples were separated by SDS-PAGE, transferred to nitrocellulose membranes, and detected using primary antibodies against EGFP (rabbit, in house), nsP3 of SFV or CHIKV (both rabbit, in house), β-actin (mouse, sc-47778; or goat, sc-1616; Santa Cruz Biotechnology, Dallas, TX, USA) or CD2AP (mouse, (B4) sc-25272, or rabbit, (H290) sc-9137, both Santa Cruz Biotechnology). Species-specific horseradish peroxidase (HRP) conjugated secondary antibodies (LabAs Ltd., Tartu, Estonia) and enhanced chemiluminescence reagents were used to develop the blots as described previously [9].

### 2.6. Immunofluorescence Microscopy

Cells were grown on coverslips in 35 mm dishes. At 50% confluence, the cells were infected with wt CHIKV, SFV or their mutant versions harbouring substitutions mutations in nsP3 at an MOI 10, mock-infected cells were used as a control. At selected time points, the cells were fixed with 4% paraformaldehyde for 10 min and subsequently permeabilized with 0.5% Triton X-100 for 3 min. Cells were washed with PBS, blocked with 5% horse serum in PBS, and stained for 1 h with primary antibodies against SFV, CHIKV nsP3 (both rabbit, in-house), CD2AP (mouse (B4) sc-25272; rabbit (H290) sc-9137), SH3KBP1 (mouse sc-166862, Santa Cruz Biotechnologies) and/or dsRNA (mouse J2, Scicons, Szirák, Hungary). Incubation with primary antibody was followed by incubation with secondary anti-mouse or anti-rabbit antibodies conjugated to Alexa Fluor555 or Alexa Fluor488 (Thermo Fisher Scientific). Draq5 (BioStatus, Loughborough, UK) was used to counterstain nuclei. Washed coverslips were mounted in mounting medium and images were obtained and analysed using a LSM710 confocal microscope (Zeiss, Oberkochen, Germany) or TCS SP5 X microscope (Leica, Wetzlar, Germany) equipped with a super continuum pulsed white laser. Images were processed using Photoshop (Adobe Systems Incorporated, San Jose, CA, USA).

## 3. Results

### 3.1. CHIKV nsP3 Hypervariable Domain Binds CD2AP and Other Host Proteins

**Figure 1:**
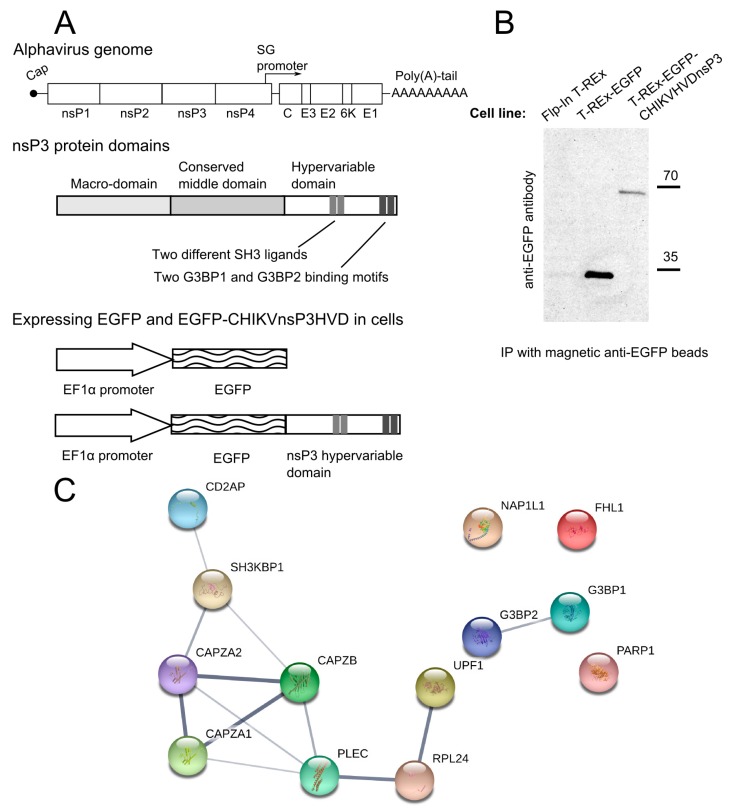
Proteins identified to associate with CHIKV nsP3 HVD. (**A**) The organization of CHIKV genome, the domains of nsP3 and schematics of constructs for expressing EGFP-CHIKVnsP3HVD and non-tagged EGFP in Flp-In T-REx cells; (**B**) Immunoblot analysis using anti-EGFP antibodies and immunoprecipitated (IP) samples obtained from the parental Flp-In T-Rex cells and transgenic T-REx-EGFP and T-REx-EGFP-CHIKVnsP3HVD cell lines; (**C**) Interaction network of proteins associated with the HVD of CHIKV nsP3 was created using STRING database; for this analysis proteins that showed at least 2-fold enrichment in SILAC-based quantitative assay were chosen.

The HVD of CHIKV nsP3 (CHIKVnsP3HVD) was fused to the C-terminus of EGFP and the sequence encoding this chimeric protein was placed under the control of human EF-1α promoter. Cell line was established using Flp-In T-REx 293 cells and the chimeric protein expressed continuously. Obtained cell line was designated T-REx-EGFP-CHIKVnsP3HVD. Similarly, a control cell line T-REx-EGFP that expresses EGFP alone was obtained (Figure 1A). Both cell lines stably expressed the intended protein. However, the expression level of EGFP-CHIKVnsP3HVD was found to be lower than that of non-tagged EGFP (Figure 1B); the effect could have resulted from the presence of a degradation signal located at the very end of nsP3 HVD [39].

Stable isotope labelling with amino acids in cell culture (SILAC) based quantitative proteomics strategy [55] that allows discriminating non-specifically captured proteins from real interactors was chosen to identify host proteins that bind nsP3 HVD of CHIKV. In the forward experiment T-REx-EGFP-CHIKVnsP3HVD cells were grown in a medium containing heavy lysine and arginine and T-REx-EGFP cells were grown in a medium with light amino acids; in the reverse experiment, the growth media were switched. Three biological replicates were obtained: twice using forward and once reverse setup. Following immunoprecipitation via EGFP binding magnetic beads, samples from both cell lines were mixed and subjected to analysis by mass-spectrometry. At least two peptides were used to identify a cellular protein; proteins were considered to bind CHIKV nsP3HVD if they were on average at least two times more abundant in the sample obtained from T-REx-EGFP-CHIKVnsP3HVD cells, compared to the control sample obtained from T-REx-EGFP cells. Multiple proteins with diverse known functions were identified using this approach (Supplementary Table S1); most of them were represented by proteins involved either in RNA metabolism or being components of cytoskeleton (Figure 1C). Among the proteins identified were some previously known interactors of alphavirus nsP3 HVD, including G3BP1, G3BP2 and PARP1 [7,35,43,56], serving as positive control for the procedure. Binding of cytoskeleton modulator CD2AP, previously shown to interact with HVD of nsP3 of VEEV and EEEV [7,10], was found to be the most evident. In addition to CD2AP itself, its homologue SH3KBP1 (also known as CIN85), and their known interaction partners F-actin-capping protein subunit alpha-1 (CAPZA1), F-actin-capping protein subunit alpha-2 (CAPZA2) and F-actin-capping protein subunit beta (CAPZB) [57] were also identified as proteins associated with HVD of CHIKV nsP3 (Figure 1C). As CD2AP showed the highest enrichment in samples containing CHIKV nsP3 HVD, it was chosen for further studies to reveal its role in CHIKV infection (Supplementary Table S1).

**Figure 2:**
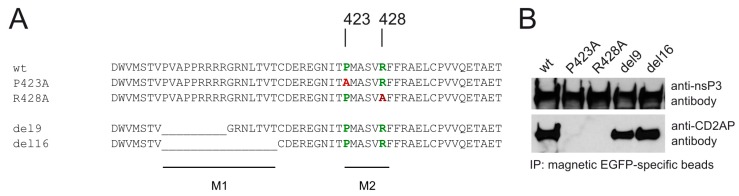
Mapping of CD2AP-binding site in HVD of CHIKV nsP3. (**A**) Sequences of two consecutive potential SH3 domain ligands (M1, M2) in HVD of CHIKV nsP3 (wt) and positions of the mutations that were introduced into EGFP CHIKVnsP3HVD expression constructs: Δ9, Δ16, P423A, R428A, Δ16 + P423A, Δ16 + R428A; “_” designates deleted residue; (**B**) Flp-In T-REx cells were transfected with plasmids expressing CHIKVnsP3HVD harbouring mutations indicated above the panel; 24 h later immunoprecipitation (IP) via EGFP-capturing beads was carried out, obtained samples were analysed by immunoblot for the presence of CD2AP and EGFP CHIKVnsP3HVD.

CD2AP has three SH3 domains [57], thus, it was initially hypothesized that the binding of CD2AP to HVD of CHIKV nsP3 occurs via the proline and arginine rich element (398-PVAPPRRRRGNLTVTC-414, hereafter referred to as M1 (for motif 1, Figure 2A)) which resembles SH3 domain ligand (Figure 2A). This element in nsP3 HVD of SFV has been previously shown to interact with Amphiphysin1 and Amphiphysin2, both have a single SH3 domain [50]. Of note, we were not able to identify amphiphysins as interactors of HVD of CHIKV using the approach described above (Figure 1, Supplementary Table S1) possibly because the experimental conditions were not suitable for detection of such interaction. Two deletions were introduced into CHIKV nsP3 HVD: the first removing the N-terminal 9 amino acid residues of M1 (Δ9) and the second removing all 16 amino acid residues (Δ16) of M1 (Figure 2A). Surprisingly, as evidenced by results of co-immunoprecipitation (co-IP) experiment, neither of these deletions had detectable effect on the interaction of nsP3 with CD2AP (Figure 2B). Further sequence analysis indicated the presence of another potential SH3 ligand for CD2AP [58], with the sequence 423-PMASVR-428 (hereafter referred to as M2), in HVD of CHIKV nsP3 (Figure 2A). Mutation of either the proline or arginine residues in the M2 motif to alanine (P423A or R428A) resulted in complete loss of interaction with CD2AP (Figure 2B), which clearly establishes the crucial role of M2 in the interaction with CD2AP.

### 3.2. CD2AP Interacts with HVD of nsP3 from Many, but Not All Alphaviruses

**Figure 3:**
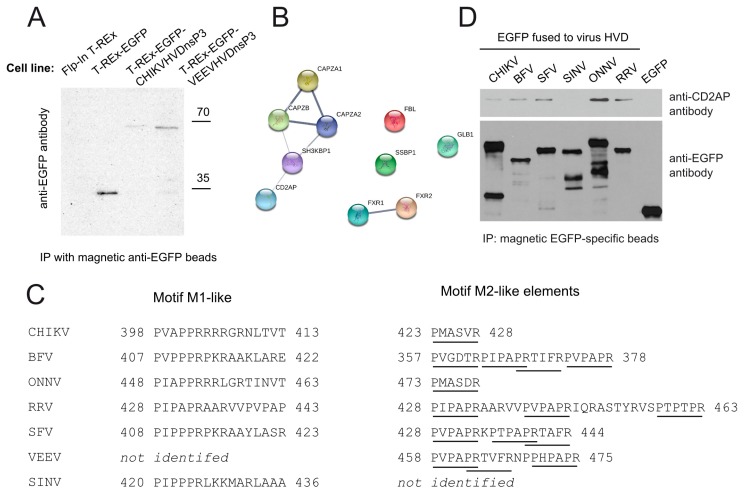
Binding of CD2AP by nsP3 HVDs of different alphaviruses. (**A**) Western blot analysis of immunoprecipitated samples obtained from Flp-In T-Rex, T-Rex-EGFP, T-Rex-EGFP-CHIKVnsP3HVD and T-Rex-EGFP-VEEVnsP3HVD cells with anti-EGFP antibody; (**B**) Network analysis of cellular proteins captured by VEEV nsP3 HVD performed as described for Figure 1C; (**C**) M1 and M2 like elements identified via multiple sequence alignment in nsP3 HVD of different Old and New World alphaviruses. Underline indicates CHIKV CD2AP-binding M2 motif identified in this study or a motif similar to it; (**D**) Flp-In T-REx cells were transfected with plasmids expressing EGFP fused to HVD from the indicated Old World alphaviruses; 24 h p.t. cells were lysed and EGFP-specific magnetic beads were used to pull down proteins; obtained samples were probed for presence of CD2AP using antibody against CD2AP. Blot developed using anti-EGFP antibody is shown as recombinant protein expression/capture control.

New and Old World alphaviruses have different mechanisms for interaction with host cells, e.g., for suppression of antiviral responses. Furthermore, it has recently been demonstrated that nsP3 proteins of New and Old World alphaviruses interact with different sets of host proteins [7,10]. To compare the binding partners of nsP3 HVD of New and Old World alphaviruses, we created another Flp-In T-REx 293 cell line that expresses EGFP fused with HVD of the VEEV nsP3 protein, hereafter referred to as T-Rex-EGFP-VEEVnsP3HVD (Figure 3A). By conducting above described SILAC-based proteomics experiment it was identified that HVD of VEEV nsP3 does, similarly to its counterpart from CHIKV, interact with numerous cellular proteins including proteins linked to RNA metabolism or cytoskeleton (Figure 3B, Supplementary Table S1). As expected, several previously described and characterized interaction partners of VEEV nsP3, including FXR1 and FXR2 [7], were identified. As expected, CD2AP was also found to interact with VEEV nsP3 HVD confirming that unlike stress granule proteins, interaction with CD2AP is not limited to either Old or New World alphaviruses.

Sequence analysis revealed that the HVD of VEEV nsP3 contains the sequence 458-PVPAPRTVFRNPPHPAPR-475 that consists of three consecutive/overlapping M2-like motifs. Furthermore, alignment of sequences of nsP3 HVD from different alphaviruses revealed that majority of them—SFV, Barmah Forest virus (BFV), Ross River virus (RRV), O’nyong’nyong virus (ONNV), VEEV—have an M2 motif; as an exception it is not present in nsP3 of SINV (Figure 3C). This is in accordance with a recent publication in which CD2AP was not detected among the binding partners of SINV nsP3, in contrast to nsP3 of VEEV and CHIKV [10].

Similar to VEEV, three consecutive or overlapping M2-like motifs (428-PVPAPRKPTPAPRTA FR-444) are present in HVD of SFV (Figure 3C). For different viruses, the order of M1 and M2 is generally the same as in CHIKV except that in nsP3 of BFV the potential M2 motifs are located upstream of M1. To test if the binding of CD2AP corresponds to the results of sequence analysis, the nsP3 HVDs from above listed alphaviruses were fused to the C-terminus of EGFP and transiently expressed in Flp-In T-REx cells. The co-immunoprecipitation experiment revealed that, as predicted, HVDs of SFV, BFV, ONNV, and RRV all interacted with CD2AP, while HVD of SINV nsP3 did not (Figure 3D). This data suggests that M2 acts as binding site of CD2AP for different alphaviruses. It also confirmed that while members of both New and Old World alphaviruses can interact with CD2AP, this interaction is not universal as at least one virus, SINV, was demonstrated to lack this interaction.

### 3.3. CD2AP Binding Motif Mediates nsP3 HVD Interaction with Multiple Cellular Proteins

CD2AP is known to complex with other cytoskeleton proteins [57,59] (Figure 1C), among them its homolog SH3KBP1. Similar to CD2AP, SH3KBP1 has three SH3 domains and is likely to bind directly to the M2 motif of nsP3 as well. To study the matter by using SILAC-based proteomics, we first attempted to construct a stable cell line expressing EGFP tagged with CHIKV HVD with P423A + R428A substitutions in M2. However, for unknown reasons, our repeated attempts to obtain such a cell line were not successful. Therefore, we took advantage of the finding that SFV has similar M2 motifs (Figure 3C) and that its nsP3 HVD binds to CD2AP similarly to CHIKV (Figure 3D). As the M2 motif in HVD of SFV nsP3 is triplicated, six point mutations (P428A, R433A, P435A, P439A, R440A, and R444A) were introduced to knock out all of them; hereafter simultaneous mutations of all P and R in M2 will be referred to as mutM2. Next, cell line expressing EGFP tagged with SFV HVD and, importantly, cell line expressing EGFP tagged with SFV HVD-mutM2 were obtained. We compared these cell lines to each other using the SILAC-based quantitative proteomics approach and identified difference in binding partners for wt and mutant protein. As expected, mutM2 substitutions had no significant effect on the binding of G3BP proteins, which bind motifs close to the C-terminus of nsP3 (Table 1, Supplementary Table S1). In sharp contrast, the analysis clearly revealed that mutation of M2 in the HVD resulted in loss of ability of nsP3 of SFV to interact with CD2AP and SH3KBP1. In addition, CAPZA1, CAPZA2 and CAPZB (Table 1, Supplementary Table S1) also did not co-precipitate with nsP3 harbouring mutM2 mutation. This data confirm that M2 motif is crucial for CD2AP and SH3KBP1 binding and that in its absence several other interaction partners are lost as well.

### 3.4. Mutations in M2 Motif of nsP3 Affects RNA Infectivity and Replication of Alphaviruses

**Figure 4:**
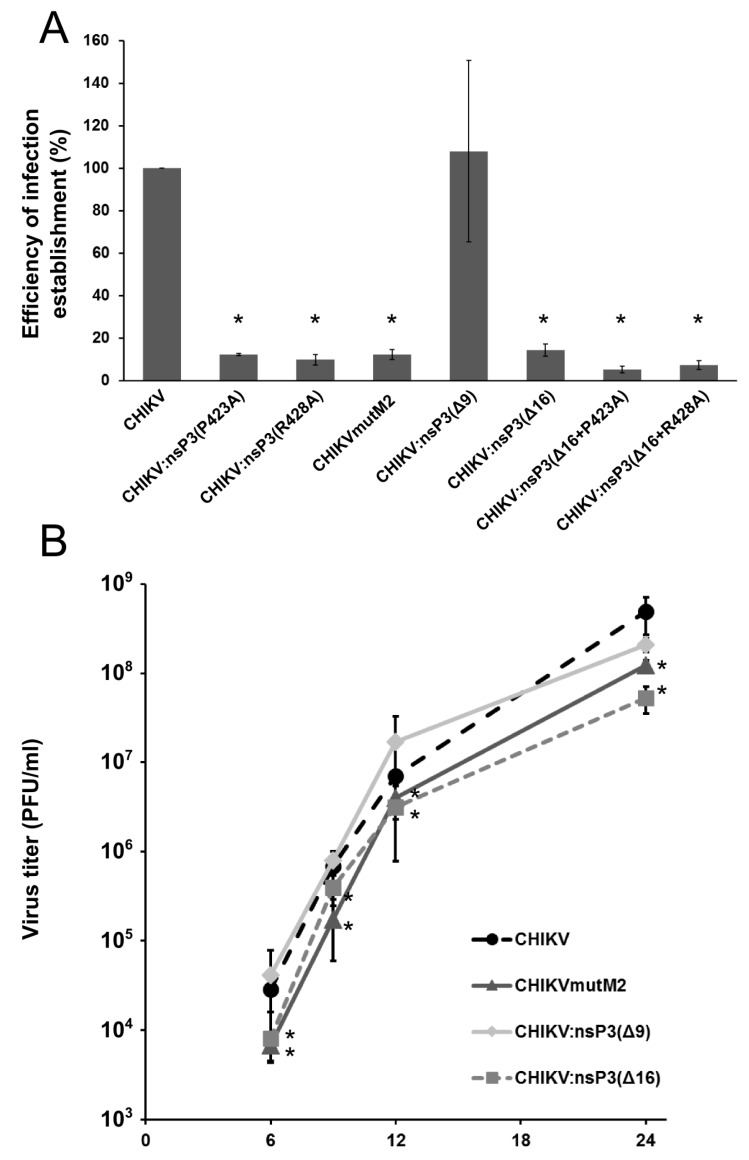
Effects of mutations in SH3 domain ligands on CHIKV RNA infectivity and CHIKV replication. (**A**) BHK-21 cells were electroporated with 1 μg of in vitro transcribed RNAs of CHIKV (wt or bearing mutation in nsP3 HVD); the infectivity of the transcripts was analysed by ICA. Mutant virus RNA infectivity is presented as a percentage of wild-type virus RNA, mean values together with standard error of three independent experiments are shown; (**B**) Growth curve of wt CHIKV or its mutant variants in BHK-21 cells infected at MOI of 0.1. Mean values of four replicates with standard deviation are shown. * denotes *p* < 0.05 (according to Student’s *t*-test), in comparison with wt virus.

To detect the importance of M2 motif and nsP3-CD2AP and/or nsP3-SH3KBP1 interactions for alphavirus replication, nsP3(P423A), nsP3(R428A) and nsP3(P423A and R428A, referred to again as mutM2) substitutions in the motif M2 were introduced in the CHIKV genome. In addition, recombinants harbouring deletions in the M1 motif (Δ9 and Δ16) or combinations of above listed mutations (Δ16 + P423A and Δ16 + R428A) were constructed and analysed. First, the RNA infectivity was analysed using infectious centre assay (ICA). It was found that the deletion of full M1 (CHIKV:nsP3(Δ16)) resulted in approximately 7-fold drop of plaque-forming ability; in contrast Δ9 mutation had no effect (Figure 4A). The fact that deletion of polyproline-rich part in M1 (Δ9) did not affect virus replication, unlike deletion of the whole M1 (Δ16), shows that seven following amino acids (407-GRNLTVT-413) could be part of an important short linear interaction motif (SLIM). Mutations in the M2 motif (CHIKV:nsP3(P423A), CHIKV:nsP3(R428A) and CHIKVmutM2 (P423A and R428A)) all resulted in approximately 10-fold reduction of infectious virus recovery. Further characterization showed that no significant additive effect could be detected when Δ16 mutation was combined with P423A or R428A substitutions (CHIKV:nsP3(Δ16 + P423A), CHIKVnsP3(Δ16 + R428A)) (Figure 4A). Thus, mutations in M1 and M2 motif affect CHIKV RNA infectivity and are needed for effective replication like the interaction motifs binding stress granule proteins, G3BP1 and G3BP2 [7,48,49]. The effect of M1 and M2 mutations on CHIKV replication was confirmed by comparison of virus growth curves. In BHK-21 cells, the Δ9 mutation had almost no effect on CHIKV replication, in contrast to Δ16 mutation, which reduced final virus titres approximately by one order of magnitude (Figure 4B). CHIKVmutM2 also replicated less efficiently compared to wt CHIKV; the difference in virus titres was approximately 5 to 10-fold throughout the course of the experiment (Figure 4B). The effect of mutation of SFV replication was less pronounced compared to CHIKV (Supplementary Figure S1). Overall, the results of CHIKV growth curve experiments correlated well with the results from ICA experiments. Thus, it should be concluded that motifs M1 and M2 are important for CHIKV as their deletion or mutation hampers virus replication considerably. However, unlike the situation with G3BPs [7,48], the interactions with cytoskeleton components mediated by the motif M2 are not absolutely essential for infectivity of CHIKV since the M2-mutated viruses are viable.

### 3.5. CD2AP and SH3KBP1 Interact with Viral nsP3 and Co-Localise with Replication Complexes in CHIKV and SFV Infected Cells

**Figure 5:**
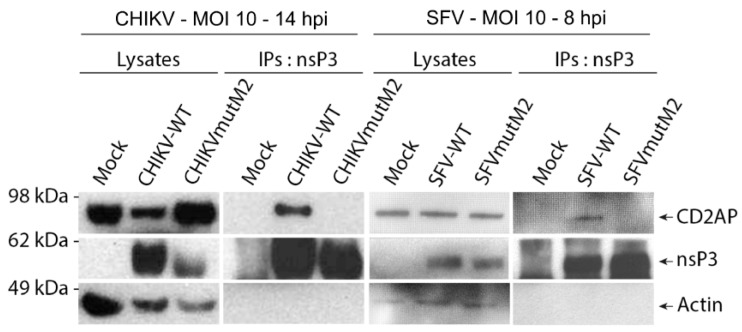
Interaction of CHIKV or SFV nsP3 with CD2AP in infected cells. HOS cells were infected with wt or mutant CHIKV or SFV at MOI of 10 and lysed at 14 (CHIKV) or 8 (SFV) hpi to carry out immunoprecipitation (IP) using antisera against nsP3 of CHIKV or SFV, respectively. Cell lysates and IP samples were probed for nsP3, CD2AP and actin.

To confirm that CD2AP binding to CHIKV and SFV nsP3 HVD is relevant in context of virus replication, human osteosarcoma cells (HOS) were infected with wt CHIKV, CHIKVmutM2, wt SFV or SFVmutM2 at MOI 10. CHIKV and SFV infected cells were lysed at 14 and 8 h post-infection (p.i.), respectively, and obtained lysates were used to carry out immunoprecipitation using antisera against nsP3 of the respective virus. As expected, CD2AP was efficiently co-immunoprecipitated with nsP3 from lysates originating from wt virus infected cells (Figure 5). However, in case of viruses harbouring substitutions in the M2 motif, CD2AP was not co-immunoprecipitated with nsP3, confirming that mutation of the motif M2 prevented interaction of nsP3 with CD2AP (Figure 5). Thus, the nsP3:CD2AP interaction is also efficiently formed in the context of virus infection.

**Figure 6:**
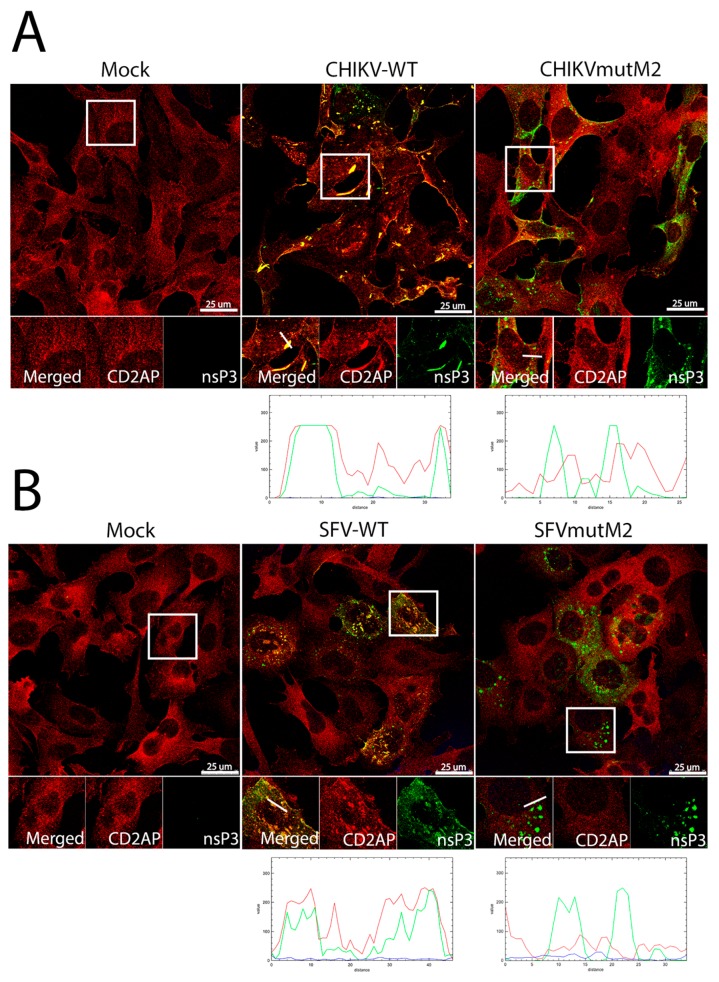
CD2AP co-localisation with CHIKV and SFV nsP3 in infected cells. HOS cells were infected with CHIKV wt or CHIKVmutM2 (**A**) or SFV wt or SFVmutM2 (**B**) at an MOI of 1. At 16 (**A**) or 8 h p.i. (**B**), cells were fixed and stained for nsP3 (green) and CD2AP (red), line scan images are given below the confocal images. Results are representative of three independent experiments.

Further, HOS cells were infected with wt or mutant CHIKV or SFV to visualize if the CD2AP and SH3KBP1 co-localise with nsP3. It was observed that in mock infected cells, CD2AP was localised rather diffusely in punctate structures in the cytoplasm while in wt CHIKV-infected cells, CD2AP was recruited to large sites of nsP3 accumulation (Figure 6A). However, consistent with proteomics data and those from co-IP experiments, in CHIKVmutM2-infected cells nsP3 no longer co-localised with CD2AP (Figure 6A). Likewise, in wt SFV-infected cells, CD2AP co-localised to a very great extent with nsP3, but remained diffusely distributed in SFVmutM2-infected cells (Figure 6B).

**Figure 7:**
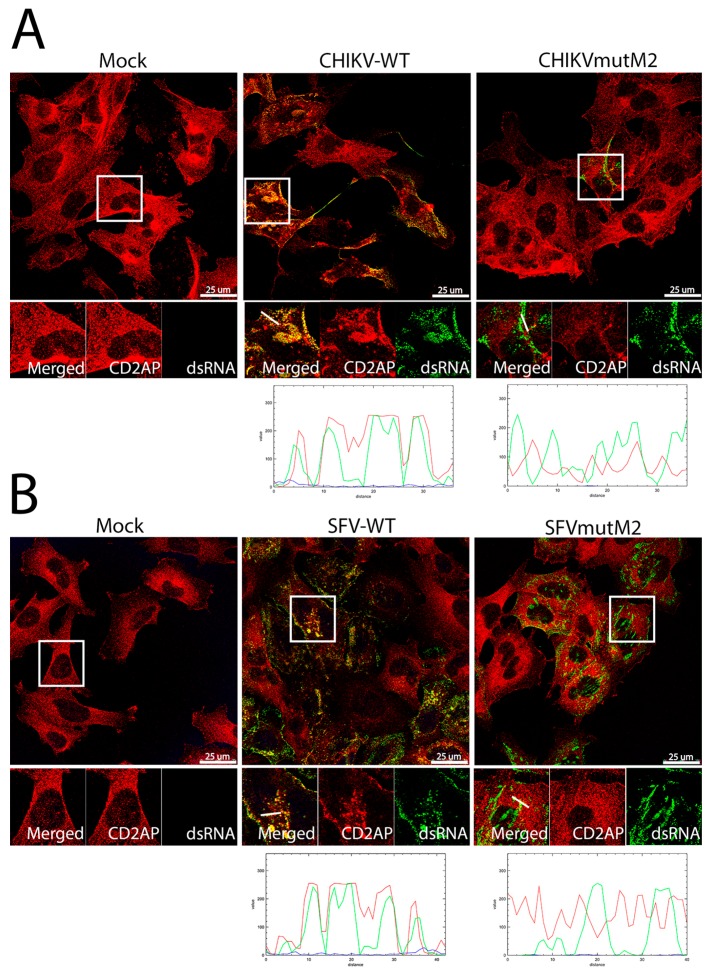
dsRNA in wt CHIKV and SFV infected cells co-localisation with CD2AP. HOS cells infected with CHIKV wt or CHIKVmutM2 (**A**) or SFV wt or SFVmutM2 (**B**) at an MOI of 1 were fixed at 16 (**A**) or 8 h pi (**B**). Thereafter the cells stained for dsRNA (green) and CD2AP (red), line scan image is given below. Results are representative of three independent experiments.

In alphavirus-infected cells only a fraction of nsP3 is incorporated into replicase complexes while the rest is present in form of different inclusions [60]. Therefore, the co-localisation of CD2AP with replication complexes was specifically analysed by co-staining the cells for CD2AP and viral dsRNA (Figure 7). It was found that CD2AP did co-localise with dsRNA in cells infected with wt CHIKV but not in CHIKVmutM2 infected cells (Figure 7A).

Though the interaction with CD2AP was lost, it did not greatly alter the localisation of replication complexes inside the cell. Similarly, nsP3 of wt SFV co-localised with CD2AP in intracellular RNA replication complexes. In contrast, dsRNA staining in cells infected with SFVmutM2 did not co-localise with CD2AP and, compared to wt SFV infected cells, dsRNA was found to be accumulated in larger clusters (Figure 7B). These data show that M2-motif mediated interaction occurs in infected cells and is a common feature for both, CHIKV and SFV.

**Figure 8:**
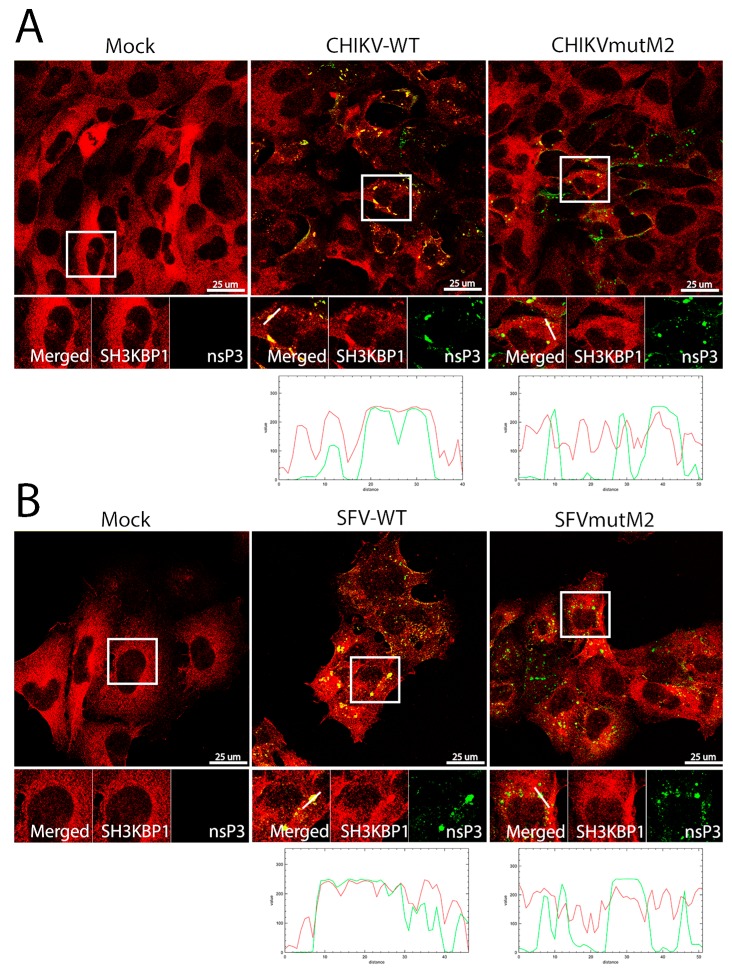
SH3KBP1 co-localises with wt CHIKV and SFV nsP3 protein. HOS cells were infected with CHIKV wt or CHIKVmutM2 (**A**) or SFV wt or SFVmutM2 (**B**) at an MOI of 1. At 16 (**A**) or 8 h pi (**B**), cells were fixed and stained for nsP3 (green) and SH3KBP1 (red) and line scan images are given. Results are representative of two independent experiments.

The related protein SH3KBP1 was also shown to interact with the HVD of CHIKV and SFV (Figure 1, Supplementary Table S1). We also analysed whether this protein is recruited to alphavirus replication complexes. In mock-infected cells SH3KBP1 was dispersed diffusely in the cytoplasm like CD2AP (Figure 8A,B). However, in cells infected with wt CHIKV or SFV it gathered into clusters that also stained for nsP3. This phenomenon did not happen in CHIKVmutM2 or SFVmutM2 infected cells, confirming that the motif M2 is also important for binding and redistributing SH3KBP1.

## 4. Discussion

Recent studies have changed the status of alphavirus nsP3 from an enigmatic component to that of a protein with many important roles [52]. For example, it is involved in removing mono(ADP-ribosyl)ation from host proteins counteracting an immune response and regulating viral RNA synthesis [38]. In addition, the nsP3 hypervariable and presumably unstructured C-terminal region has turned out to be a hub for various host-virus interactions. Some of these are not crucial for virus replication and therefore nsP3 can tolerate significant modifications in its HVD up to almost complete deletion or replacement with heterologous protein sequence [7,35,42,45,56]; it also tolerates insertion of protein-tags in several positions in the HVD. On the other hand, some interactions, such as those with stress granule proteins are crucial, possibly due to their primary role in allowing the assembly of functional replication complexes [7,46,48]. Since the HVD is intrinsically unstructured, these interactions are likely mediated by relatively short linear sequence motifs located in HVD. Indeed, multiple sequence alignment allows to detect common motifs shared between HVD of different alphaviruses even though the lengths and especially the sequences of this region vary greatly [7,39,47,50].

In this study, we expressed nsP3 HVD domains of different alphaviruses as fusion proteins with EGFP, generated several stable human cell lines and used these to capture and identify cellular protein interactors of CHIKV, VEEV and SFV HVD. The dataset we obtained for CHIKV and VEEV nsP3 HVD showed good overlap with datasets published previously, even though those were obtained using different approaches. In these previous studies, BHK-21 or Vero cells were used, either infected with SINV expressing EGFP-tagged nsP3 [43,44] or alternatively, infected with alphavirus or replicons expressing EGFP-fused HVD from a subgenomic promoter [7,10]. The good overlap serves as validation of the approach used in this study and our dataset also confirms the previous findings. We were also able to identify several proteins interacting with CHIKV nsP3 HVD, for example, PARP1, previously shown to bind nsP3 HVD of SINV [56], also G3BP1 and G3BP2 [7,47].

The roles of many of the nsP3 interacting proteins, identified in this and other studies, for alphavirus infection remain poorly understood. Among the proteins with an undescribed role in virus infection, CD2AP was one of the most prominent, at least when judged by its relative abundance in our CHIKV and VEEV nsP3 HVD interactors dataset. Further analysis using immunoblotting readily confirmed its interaction with CHIKV nsP3 HVD and allowed mapping of the interaction site; we confirmed that the binding occurs via a previously uncharacterized SLIM, termed M2. Immunofluorescence studies showed that in CHIKV-infected cells, CD2AP co-localised with nsP3 and dsRNA, and that its interaction with virus replication complex components was solely dependent on the motif M2 located in nsP3. Motifs similar to M2 were also found to be present in the nsP3 HVD of many other alphaviruses, and more importantly, experiments confirmed their nsP3 HVD interaction with CD2AP. The motif was also essential for binding to SH3KBP1, a related protein to CD2AP. Interestingly, the prototype member of genus alphavirus, SINV, lacks this motif as well as the ability of its nsP3 to interact with CD2AP, confirming findings recently reported by others [10]. The implications of this unique situation with SINV are not known; either this virus uses some other interactor(s) to substitute CD2AP or its replication does not require or benefit from this kind of interaction partner. Indeed, VEEV nsP3 does not bind G3BP1 and G3BP2 but FMR1, FXR1, FXR2 proteins instead and these interactions have been shown to be essential for virus replication [7,10]. Our study did reveal that nsP3 interaction with CD2AP and SH3KBP1 is indeed important for alphaviruses, and although both SFV and CHIKV tolerate mutations abolishing these interactions relatively well, it results in reduction of CHIKV multiplication. Similarly, motif M1 in the current that has SH3 ligand (398-PVAPPRRR-406) is known to bind Amphiphysin-1 and Amphiphysin-2, which have membrane-bending properties, abolishing this interaction results in attenuated SFV [50]. Our methodology was more suitable to capture cytoplasmic proteins, as evidenced by our failure to capture amphiphysins. The close vicinity of two SH3 ligands (in M1 and M2) may indicate that they are also functionally linked, a matter for further studies.

CD2AP is one of the organizers of cellular actin cytoskeleton [57,59]. One of the roles of CD2AP is to cap actin fibres together with CAPZA1, CAPZA2, CAPZB proteins. CD2AP and SH3KBP1, which is homologous to CD2AP, play versatile role, involved in dynamic actin cytoskeleton remodelling. These functions include recruitment of above listed actin capping proteins, it can facilitate branched actin filament formation [61] and has actin bundling properties [57,59]. Also CD2AP and SH3KBP1 have been shown to be important for receptor-mediated endocytosis and endosome formation [57,62]. However, when the motif was mutated in SFV or CHIKV we could not detect markedly altered replication complex localisation in the cells, however, there is a possibility that the lack of such interactions leads to altered replication complex stability. It is important to note that CD2AP and SH3KBP1 are likely not the only cellular proteins for which binding is mediated by motif M2. Other cytoskeleton components were also pulled down by CHIKV and VEEV nsP3 HVD, among them were also actin capping proteins CAPZA1, CAPZA2, CAPZB. Their interaction with the HVD was lost when mutations preventing interaction with CD2AP were introduced into SFV nsP3, indicating that these proteins interact with the same motif and/or with each other. Therefore, it would be important to elucidate their individual or combined roles during alphavirus infection. However, multiple protein partners prevent simple and straightforward interpretation of the data, e.g., assigning all discovered effects solely to the CD2AP binding. It is more likely that CD2AP and SH3KBP1 directly bind to nsP3 motif M2 via their SH3 domain; this domain is missing in CAPZA1, CAPZA2 and CAPZB, their binding may be mediated by other proteins including CD2AP and/or SH3KBP1. The possible functional redundancy also severely limits use of CRISPR-Cas knock-out technology as simultaneous knock-out (or even knock-down) of such a large number of host genes is challenging.

Also, it is interesting that VEEV nsP3 HVD binds the same set of cytoskeleton proteins as CHIKV and SFV. However, it is not clear if these interactions have the same role in VEEV infection as for CHIKV. It is interesting to mention that while our analysis revealed CD2AP, SH3KBP1, CAPZB, CAPZA1 and CAPZA2 as top interaction partners both for CHIKV and VEEV, a recent study found them to interact more prominently with nsP3 of VEEV; in fact CAPZA1 and CAPZA2 were not detected as interaction partners for nsP3 of CHIKV in that study [10]. The reasons for this discrepancy can be attributed to the use of a different cell line and/or different protein capture approaches. However, it may also indicate that interaction with this set of proteins is more prominent (or more stable) and therefore possibly more important for VEEV. Thus, it is an important topic for further studies to control if the replication of New World alphaviruses is dependent on their ability to bind CD2AP and the other cytoskeleton components.

It should also be emphasized that while this study focused mostly on one motif in nsP3 HVD, there are potentially other still uncharacterized motifs in HVD that are involved in interactions with host proteins, for example the seven last amino acids in the M1 (407-GRNLTVT-413) of CHIKV. This generates a possibility for the presence of a complicated network, where the effect of one virus-host interaction is likely modified by the presence or absence of several other interactions. Unravelling this network, revealing functional significance of such interactions and their combinations not only for virus replications in cell culture but also in vivo, represent significant challenges for additional studies. Due to the flexibility of nsP3 HVD it can act as an evolutionary “playground”—new motifs to bind new partners can easily evolve, increasing the chances that the virus can evade restricting factors. Additionally, the results of studies of SLIMs can give valuable information to design better vaccine candidates [63] as mutating those motifs can help to produce weakened viruses.

## Figures and Tables

**Table 1:** Motif M2 of SFV nsP3 HVD associates with multiple proteins.

Protein Name	Fold Difference in the Amounts Host Proteins Captured by HVD and HVD-mutM2 of SFV nsP3
SH3KBP1	11.0
CD2AP	8.1
CAPZB	4.7
CAPZA1	4.7
CAPZA2	2.6
G3BP1	0.9
G3BP2	0.8

## Supplementary Materials

The following are available online at http://www.mdpi.com/1999-4915/10/5/226/s1, Table S1: Number of peptides identified per protein. Figure S1: Growth curve of wt SFV or its mutant SFVmutM2 in BHK-21 cells infected at MOI of 1.
