# Supplementary figures and images for: Mutation of CD2AP and SH3KBP1 Binding Motif in Alphavirus nsP3 Hypervariable Domain Results in Attenuated Virus
Source: Viruses. 2018 Apr 27;10(5):226. doi: 10.3390/v10050226 (PMC5977219; doi:10.3390/v10050226)

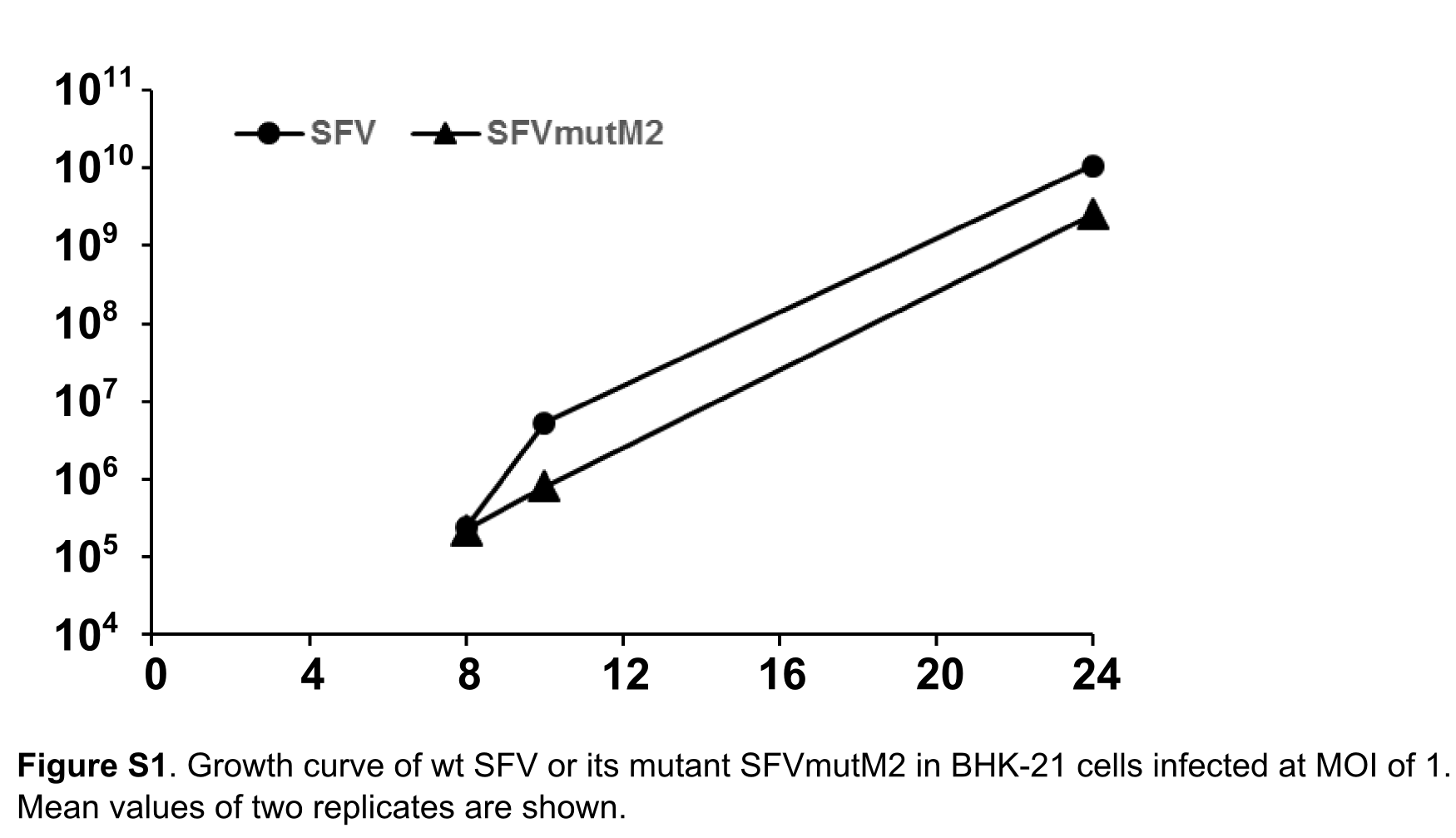

Supplement: Supplementary file 1 [file viruses-10-00226-s001.zip › FigS1.tif]
